# Design, Implementation, and Outcomes of a Volunteer-Staffed Case Investigation and Contact Tracing Initiative at an Urban Academic Medical Center

**DOI:** 10.1001/jamanetworkopen.2022.32110

**Published:** 2022-09-23

**Authors:** Rachel Feuerstein-Simon, Katherine M. Strelau, Nawar Naseer, Kierstyn Claycomb, Austin Kilaru, Hannah Lawman, Lydia Watson-Lewis, Heather Klusaritz, Amelia E. Van Pelt, Nadia Penrod, Tuhina Srivastava, Hillary C.M. Nelson, Richard James, Moriah Hall, Elaine Weigelt, Courtney Summers, Emily Paterson, Jaya Aysola, Rosemary Thomas, Deborah Lowenstein, Preeti Advani, Patricia Meehan, Raina M. Merchant, Kevin G. Volpp, Carolyn C. Cannuscio

**Affiliations:** 1 Department of Family and Community Health, Perelman School of Medicine, University of Pennsylvania, Philadelphia; 2 Center for Public Health Initiatives, University of Pennsylvania, Philadelphia; 3 Biomedical Graduate Studies, Perelman School of Medicine, University of Pennsylvania, Philadelphia; 4 Department of Microbiology, Perelman School of Medicine, University of Pennsylvania, Philadelphia; 5 Leonard Davis Institute of Health Economics, University of Pennsylvania, Philadelphia; 6 Center for Emergency Care Policy and Research, Perelman School of Medicine, University of Pennsylvania, Philadelphia; 7 Department of Emergency Medicine, Perelman School of Medicine, University of Pennsylvania, Philadelphia; 8 Philadelphia Department of Public Health, Philadelphia, Pennsylvania; 9 Now with Novo Nordisk, Plainsboro, New Jersey; 10 Center for Health Incentives and Behavioral Economics, University of Pennsylvania, Philadelphia; 11 Center for Global Health, Perelman School of Medicine, University of Pennsylvania, Philadelphia; 12 Penn Institute for Biomedical Informatics, Perelman School of Medicine, University of Pennsylvania, Philadelphia; 13 School of Nursing, University of Pennsylvania, Philadelphia; 14 Department of Medical Ethics and Health Policy, Perelman School of Medicine, University of Pennsylvania, Philadelphia; 15 Department of Medicine, Perelman School of Medicine, University of Pennsylvania, Philadelphia; 16 Center For Health Equity Advancement, Perelman School of Medicine, University of Pennsylvania, Philadelphia; 17 Perelman School of Medicine, University of Pennsylvania, Philadelphia; 18 Center for Health Equity Research and Promotion, Corporal Michael J. Crescenz Department of Veterans Affairs Medical Center, Philadelphia, Pennsylvania; 19 Department of Health Care Management, Wharton School, University of Pennsylvania, Philadelphia

## Abstract

**Importance:**

The COVID-19 pandemic has claimed nearly 6 million lives globally as of February 2022. While pandemic control efforts, including contact tracing, have traditionally been the purview of state and local health departments, the COVID-19 pandemic outpaced health department capacity, necessitating actions by private health systems to investigate and control outbreaks, mitigate transmission, and support patients and communities.

**Objective:**

To investigate the process of designing and implementing a volunteer-staffed contact tracing program at a large academic health system from April 2020 to May 2021, including program structure, lessons learned through implementation, results of case investigation and contact tracing efforts, and reflections on how constrained resources may be best allocated in the current pandemic or future public health emergencies.

**Design, Setting, and Participants:**

This case series study was conducted among patients at the University of Pennsylvania Health System and in partnership with the Philadelphia Department of Public Health. Patients who tested positive for COVID-19 were contacted to counsel them regarding safe isolation practices, identify and support quarantine of their close contacts, and provide resources, such as food and medicine, needed during isolation or quarantine.

**Results:**

Of 5470 individuals who tested positive for COVID-19 and received calls from a volunteer, 2982 individuals (54.5%; median [range] age, 42 [18-97] years; 1628 [59.4%] women among 2741 cases with sex data) were interviewed; among 2683 cases with race data, there were 110 Asian individuals (3.9%), 1476 Black individuals (52.7%), and 817 White individuals (29.2%), and among 2667 cases with ethnicity data, there were 366 Hispanic individuals (13.1%) and 2301 individuals who were not Hispanic (82.6%). Most individuals lived in a household with 2 to 5 people (2125 of 2904 individuals with household data [71.6%]). Of 3222 unique contacts, 1780 close contacts (55.2%; median [range] age, 40 [18-97] years; 866 [55.3%] women among 1565 contacts with sex data) were interviewed; among 1523 contacts with race data, there were 69 Asian individuals (4.2%), 705 Black individuals (43.2%), and 573 White individuals (35.1%), and among 1514 contacts with ethnicity data, there were 202 Hispanic individuals (12.8%) and 1312 individuals (83.4%) who were not Hispanic. Most contacts lived in a household with 2 to 5 people (1123 of 1418 individuals with household data [79.2%]). Of 3324 cases and contacts who completed a questionnaire on unmet social needs, 907 (27.3%) experienced material hardships that would make it difficult for them to isolate or quarantine safely. Such hardship was significantly less common among White compared with Black participants (odds ratio, 0.20; 95% CI, 0.16-0.25).

**Conclusions and Relevance:**

These findings demonstrate the feasibility and challenges of implementing a case investigation and contact tracing program at an academic health system. In addition to successfully engaging most assigned COVID-19 cases and close contacts, contact tracers shared health information and material resources to support isolation and quarantine, thus filling local public health system gaps and supporting local pandemic control.

## Key Points

**Question:**

How did an urban academic medical center design and implement a volunteer-staffed COVID-19 contact tracing initiative in collaboration with the local health department?

**Findings:**

In this case series of nearly 5000 health system patients and their close contacts, volunteer contact tracers played a key role in delivering infection control guidance and resources (eg, food and medication deliveries) needed for patients to safely isolate or quarantine and interrupt chains of transmission. Of 3324 participants who completed a questionnaire on unmet social needs, 27% needed assistance with securing basic resources to follow infection control guidance, and this was more common among Black than White cases and contacts.

**Meaning:**

These findings suggest that in addition to delivering outpatient and inpatient medical care, health systems may play important roles in prevention during public health emergencies working in collaboration with local health departments.

## Teaser

This case series evaluates the design, implementation, and outcomes of a volunteer-staffed contract tracing program at a large academic health center.

## Introduction

In the US, most public health functions, including case investigation and contact tracing (CI and CT), are the purview of state and local health departments (LHDs). In the years leading up to the COVID-19 pandemic, regular budget cuts and frequent employee departures resulted in a public health system often under-resourced to perform even routine duties.^1,2,3^ From 2010 to 2020, spending among state and LHDs decreased by nearly 20% per capita and the US public health workforce lost nearly 55 000 jobs.^1,3^ Furthermore, as little as 1.5% of the US government’s $3.6 trillion annual health budget is directed toward population-level public health activities.^4^ For comparison, the annual Centers for Disease Control and Prevention (CDC) budget in fiscal year 2022 was $15.4 billion, comparable to the posted 2021 operating costs of a single large private health system.^5,6^

During the COVID-19 pandemic, private institutions experienced the consequences of public health disinvestment, including the necessity to engage in activities that had traditionally been the province of public health authorities.^7,8,9,10^ For example, schools, colleges, and universities implemented a suite of mitigation measures, including hybrid learning models, symptomatic testing and asymptomatic screening, and CI and CT.^7,8^ Hollywood production crews created detailed testing plans, and many completed their own CI and CT.^9,10^ Health systems also leveraged existing infection control infrastructure and expanded their operations to investigate transmission in health care settings and sometimes in the community.^7^

This study describes a volunteer-staffed CI and CT initiative at the Perelman School of Medicine run in collaboration with the Philadelphia Department of Public Health (PDPH) that served University of Pennsylvania Health System (UPHS) patients with COVID-19 and their close contacts. We report on program design, implementation, and outcomes, as well as how the program addressed unmet social needs to facilitate isolation and quarantine. This study also identifies challenges and opportunities for private health system collaborations with LHDs.

## Methods

All program activities in this case series were approved as quality improvement by the University of Pennsylvania’s Institutional Review Board. CI and CT volunteers obtained verbal consent from all cases and contacts to participate in interviews. This study followed the relevant elements of the reporting guidelines for case series.

### Program Implementation

Team members (RFS and CCC) met with PDPH officials on March 25, 2020, and identified the need for CI and CT support (eFigure 1 in the Supplement). Initially, PDPH sought 10 volunteers to augment their in-house CI and CT capacity. Instead, due to rapidly increasing pandemic demands, our team recognized the need to build a parallel CI and CT capacity so that we could flexibly respond and integrate members into the city’s workflow as needed. Ultimately, we trained a stand-alone team to work in collaboration with PDPH with a focus on supporting patients tested within UPHS.

### Volunteer Recruitment and Screening

Between March 2020 and May 2020, volunteers were recruited through university email listservs. We offered fieldwork opportunities for students whose academic programs had been disrupted by the pandemic. Team members also received unsolicited offers to volunteer from alumni, personal contacts, and concerned community members. Candidates completed a screening survey regarding demographics, qualifications, mental health status and distress, and motivations. From March 28 to June 1, 2020, 324 interested candidates completed the survey (eFigure 2 in the Supplement). Accompanied by social workers, team members conducted telephone interviews with select candidates to identify candidates who would work well in stressful pandemic circumstances. From March 2020 to May 2021, 160 people served as volunteers.

### Team Structure

**Figure 1.**
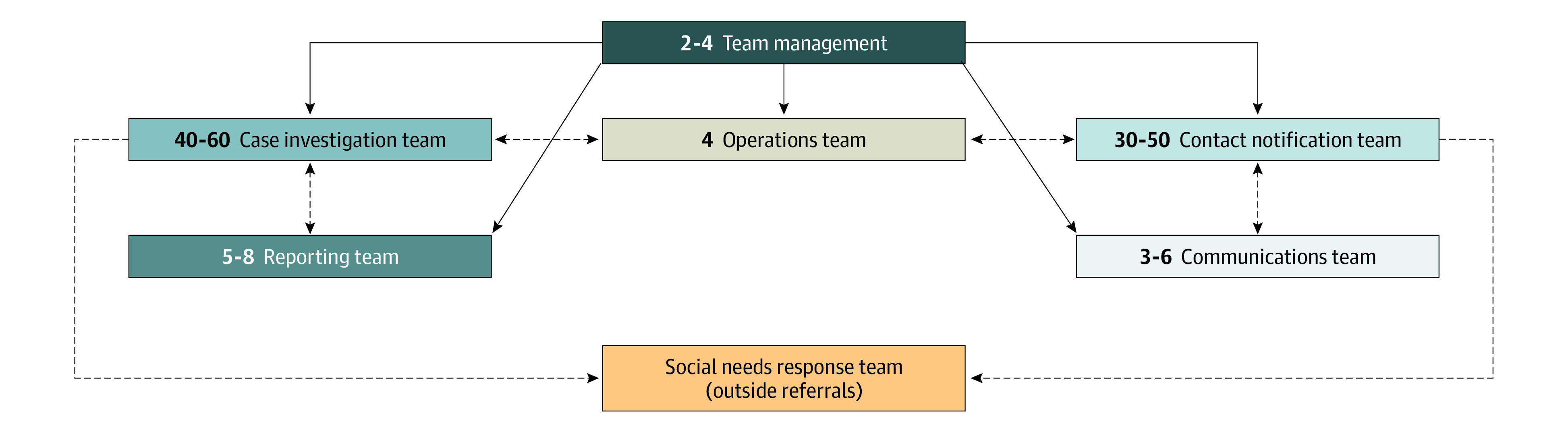
Team Structure

Volunteers were assigned to clearly delineated roles (Figure 1). In addition to CI and CT teams, specific teams addressed cases from high-risk work sites (eg, nursing homes and prisons), communications (eg, sending frequently asked question lists and employer letters) (eFigure 3 in the Supplement), and operations (eg, volunteer case and contact assignments). Team management designed, implemented, and managed the program, including supporting recruitment efforts, creating and deploying the training, coordinating with the university and local departments of public health, reporting to local and state officials, and overseeing all volunteer leaders, among other tasks. The case investigation team was responsible for investigating cases positive for SARS-CoV-2, identifying their close contacts, and delivering up-to-date isolation guidance. The contact tracing team was responsible for notifying close contacts of cases regarding their possible exposure and delivering up-to-date quarantine and testing guidance. The operations team uploaded to REDCap case information and contact details derived from case investigations and assigned calls to case investigators and contact tracers. The reporting team for high exposure risk cases reported to the relevant LHD cases who were positive and were likely unable to maintain physical distancing, or high exposure risk cases (HERCs). These included individuals living or working in congregate care settings (eg, nursing homes or shelters) or working in occupations with high likelihood of transmission to other people (eg, meat processing plants). The communications team provided follow-up guidance on safe isolation and quarantine (eFigure 3 in the Supplement) and employer letters for any case or contact who requested one. The social needs response team was an external team that connected patients to a range of services, including food delivery, rent assistance, medical transportation, and assistance identifying a primary care physician. See eMethods in the Supplement for a detailed description of team roles and responsibilities.

### Training

The team developed and led an initial synchronous virtual 2-hour training session to prepare volunteers for CI and CT, incorporating concepts and materials from US and international health departments and colleague input.^1^ Training modules included background on COVID-19 and CDC guidelines, CI and CT processes, the workflow and data collection system, and consent and interview techniques. Although volunteers received training for CI and CT, they were later divided into teams dedicated to 1 role. Volunteers who spoke with contacts had no access to names of index cases, to prevent unwitting disclosure of patient information. All volunteers completed asynchronous training on protection of human participants.^11^

### Workflow and Interview Guide

Based on a daily case log generated by UPHS personnel, CI volunteers reached out to patients who tested positive for SARS-CoV-2 at any UPHS testing location. Through structured prompts regarding activity during the infectious period, interviewers sought to identify anyone with whom patients had close contact, initially defined as being within 6 feet for more than 15 minutes. CI volunteers also delivered isolation guidance and recommendations for keeping household members safe. Then, using information obtained during CIs, volunteers from the CT team called all identified close contacts with accurate contact information, instructing contacts to quarantine for 14 days from the date of exposure and answering questions about testing. Interview guides for CI and CT drew on the team’s expertise in qualitative research, as well as tools from other contact tracing teams.^12,13^ As CDC and local public health guidance evolved, the communication team interpreted the guidance, resolved contradictory CDC and local recommendations, and created explanatory materials (eFigure 3 in the Supplement).

Participant race and ethnicity were self-reported. Options for race were African American or Black, American Indian or Alaskan Native, Asian, Native Hawaiian or Pacific Islander, White, or unspecified “other.” These categories were based on review of materials from other CI and CT interview guides. Respondents could answer yes or no to Hispanic or Latino. For demographic statistics, American Indian or Alaskan Native, Native Hawaiian or Pacific Islander, and unspecified other were combined as other because there were 0 to very few responses for these categories. Other categories were combined for unmet social needs analysis to facilitate statistical analysis due to low numbers reported. Race and ethnicity were assessed as part of a standard demographic questionnaire.

All data were collected using research electronic data capture (REDCap), a secure web-based platform,^14^ via tools that incorporated a consent script, specific questions, interview technique reminders, and isolation and quarantine guidance. REDCap tools were routinely updated to reflect continuous quality improvement and evolving public health guidance. The program incorporated safeguards to protect patient privacy, including training, continuing instruction, and restriction of data access via REDCap system design. When communicating via unsecured platforms, volunteers were cautioned not to share identifying information. CI and CT teams maintained separate REDCap projects to protect against inadvertent disclosure of patient information to contacts.

**Figure 2.**
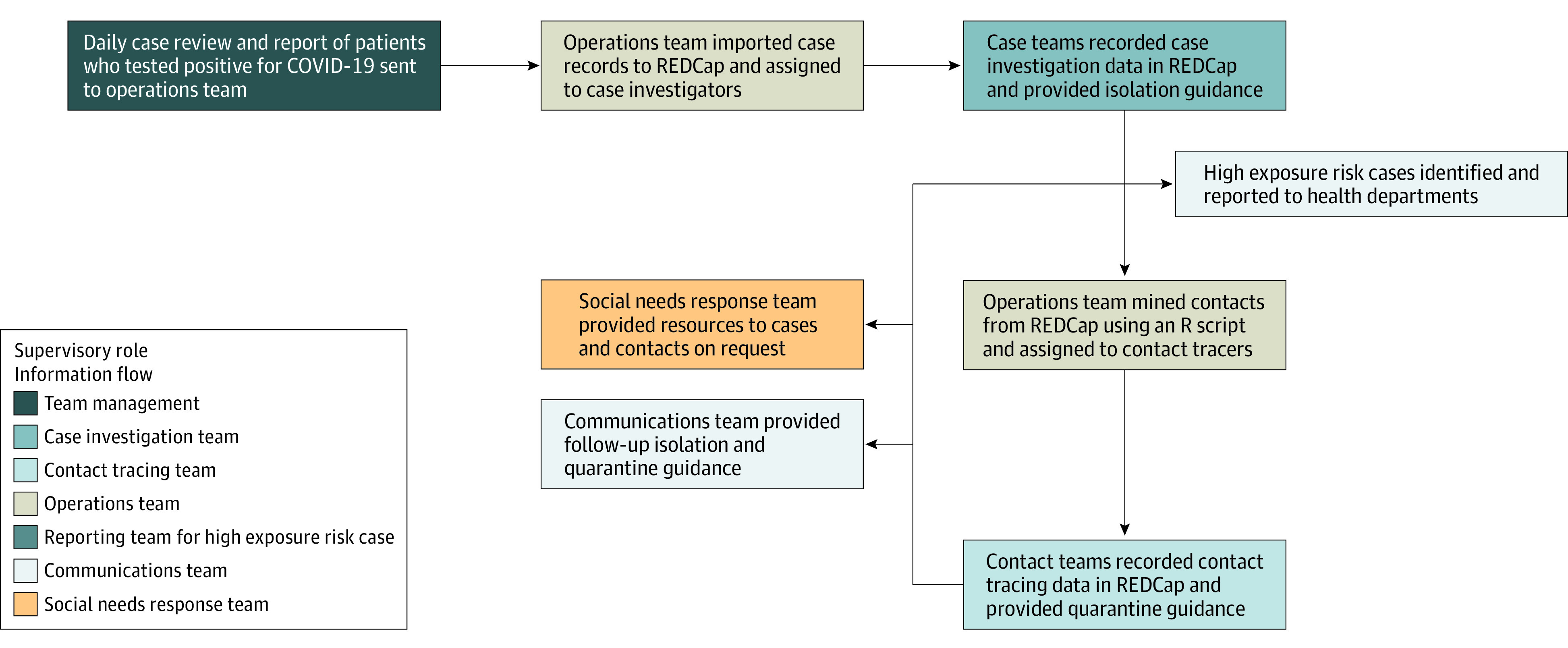
Case Investigation and Contact Tracing Operational Workflow REDCap indicates research electronic data capture.

Daily, UPHS administrators securely shared with team management a list of patients who tested positive via polymerase chain reaction (PCR) for SARS-CoV-2 in the prior 24 hours at any UPHS site (eg, emergency departments or testing sites). The operations team then assigned patients to CI and CT volunteers, with caseloads determined by volunteer availability and daily case rates. Pediatric cases and hospital inpatients were excluded because of anticipated challenges with making contact, obtaining consent, and gathering information. During surges, the team called only cases residing in Philadelphia County, although contacts were notified regardless of home address. See Figure 2 for a workflow diagram.

All team members used a mobile application that identified their outgoing calls as originating from the health system. Team members also each obtained a unique online telephone service number via Google Voice with a Philadelphia area code for return calls. On the recommendation of other programs, volunteers called each individual multiple times in rapid succession (signaling the importance of the call) at 3 different times of day over 2 days.

In April 2020, UPHS faculty created a student-staffed social needs response team (SNRT) to address unmet social needs among patients, as well as to support safe isolation and quarantine. CI and CT volunteers screened all cases and contacts for unmet social needs (eg, housing and food) and referred to the SNRT anyone who reported at least 1 unmet need and consented to referral. The REDCap form included an automated pop-up to prompt volunteers to complete a social needs referral when participants reported unmet social needs.

### PDPH Collaboration

Our team closely communicated with PDPH throughout the program. To prevent duplication of efforts, we securely shared a daily list of patients assigned to our CI volunteers. PDPH staff removed from their queue those cases who were assigned to our CI and CT volunteers. If case volume exceeded our capacity, unassigned UPHS cases were by default added to PDPH’s queue for case investigation. Information regarding contacts was not shared with PDPH unless those contacts later tested positive via PCR at a UPHS site. We also sent weekly briefing memos with programmatic updates and case and contact call summary statistics (eg, call numbers, response rates, and unmet social needs).

### Qualitative Data

Qualitative results were based on team documentation from March 2020 to May 2021, including emails, calendars, progress memos, messaging channel (Slack, Slack Technologies) conversation, and volunteer recruitment surveys. Team members reviewed these documents to synthesize programmatic challenges and successes.

### Statistical Analysis

We report outcomes of CI and CT interviews using participant data from REDCap. Descriptive statistics and odds ratios (ORs) of the likelihood of reporting social needs are reported, based on analyses conducted in R statistical software version 8.9.0 (R Project for Statistical Computing).^15^ Statistical tests were 2-sided and reported at the 95% CI.

## Results

**Figure 3.**
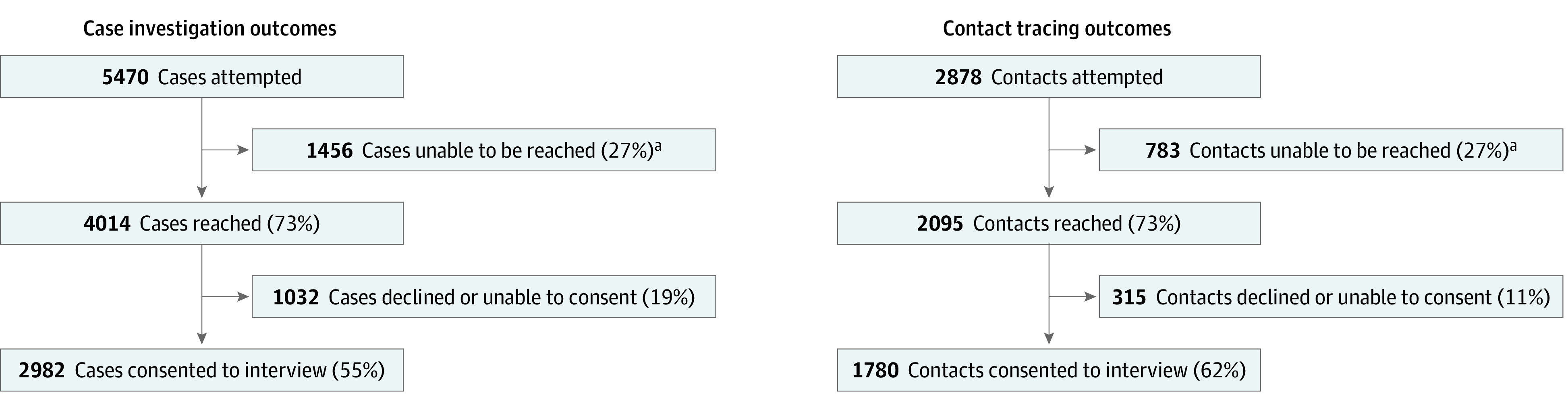
Case Investigation and Contact Tracing Call Outcomes ^a^Reasons why individuals were not reached: non-working phone number, incorrect phone number, no answer.

A large proportion of CI and CT interviews were conducted with Black individuals, women, and non-Hispanic individuals living in households of 2 to 5 people (Table 1). Of 5470 CIs attempted, nearly three-quarters of cases (4014 individuals [73.4%]) were reached. Of cases reached, 2982 individuals (74.2%) consented to be interviewed (median [range] age, 42 [18-97] years; 1628 [59.4%] women among 2741 cases with sex data) (Figure 3). Thus, 54.5% of 5470 cases were reached and consented to be interviewed. Among 2683 cases with race data, there were 110 Asian individuals (3.9%), 1476 Black individuals (52.7%), and 817 White individuals (29.2%), and among 2667 cases with ethnicity data, there were 366 Hispanic individuals (13.1%) and 2301 individuals who were not Hispanic (82.6%). Most individuals lived in a household with 2 to 5 people (2125 of 2904 individuals with household data [71.6%]). A total of 3222 unique contacts with complete contact information were reported by cases, and volunteers attempted to call 2878 of those individuals. In total, 2095 contacts who were called were reached (72.8%), and of contacts reached, 1780 individuals (85.0%) consented to participate in the interview (median [range] age, 40 [18-97] years; 866 [55.3%] women among 1565 contacts with sex data). Therefore, overall, 55.2% of contacts were interviewed. Among 1523 contacts with race data, there were 69 Asian individuals (4.2%), 705 Black individuals (43.2%), and 573 White individuals (35.1%), and among 1514 contacts with ethnicity data, there were 202 Hispanic individuals (12.8%) and 1312 individuals (83.4%) who were not Hispanic. Most contacts lived in a household with 2 to 5 people (1123 of 1418 individuals with household data [79.2%]).

**Table 1.** Self-reported Participant Characteristics

Characteristic	Participants, No. (%)	
	Cases (n = 2982)	Contacts (n = 1780)
Age, median (range)	42 (18-97)	40 (18-97)
Sex		
Total with data, No.	2741	1565
Women	1628 (59.4)	866 (55.3)
Men	1108 (40.4)	696 (44.5)
Another identity	5 (0.2)	3 (0.2)
Race		
Total with data, No.	2683	1523
African American or Black	1476 (52.7)	705 (43.2)
Asian	110 (3.9)	69 (4.2)
White	817 (29.2)	573 (35.1)
Other^a^	280 (10.0)	176 (10.8)
Ethnicity		
Total with data, No.	2667	1514
Hispanic or Latino	366 (13.1)	202 (12.8)
Not Hispanic or Latino	2301 (82.6)	1312 (83.4)
Household size, No. of occupants		
Total with data, No.	2904	1418
1	480 (16.2)	138 (9.7)
2-5	2125 (71.6)	1123 (79.2)
≥6	299 (10.1)	157 (11.1)

^a^ Includes American Indian or Alaskan Native, Native Hawaiian or Pacific Islander, or reported by participant as unspecified “other.”

In the days prior to diagnosis, cases reported engaging in a variety of activities that could expose others, including working outside the home (1113 of 2157 individuals who responded to the question [51.6%]). A minority of participants had traveled outside of the state (412 of 2736 individuals who responded to the question [15.1%]) or attended a religious gathering (51 of 2792 individuals who responded to the question [1.2%]). Despite these exposures and the likelihood that many index cases likely exposed many people, the reported number of contacts per case was low, often because cases did not have names and contact information for people they had encountered in public spaces. In addition, many cases asked us not to call exposed household members who were already infected or already quarantined.

Among 1074 cases who responded to questions regarding the type of guidance or information they received at the point of testing, nearly one-quarter (254 individuals [23.6%]) reported that they were given no instructions (eg, regarding isolation) by the site or clinician they visited for COVID-19 testing. Of those given instructions, 456 individuals (55.6%) were given verbal instructions only, 208 individuals (25.4%) were given written (print or electronic) instructions only, and 132 individuals (16.1%) were given verbal and written instructions.

Among 3324 participants who completed a screening questionnaire to identify unmet social needs that would make it difficult for them to isolate or quarantine safely, 907 (27.3%) reported at least 1 unmet social need, mostly related to difficulty paying for utilities (538 individuals [16.2%]) or paying for or obtaining food (522 individuals [15.7%]). Among participants who reported at least 1 unmet social need, most (590 individuals [65.0%]) reported 2 or more unmet social needs (17.8% overall) and 345 individuals (38.0%) reported 3 or more unmet social needs (10.4% overall) (Table 2).

**Table 2.** Reported Unmet Social Needs by Race

Unmet social need	No. of participants reporting unmet social needs, No. (%) (N = 3324)				
	Black	White	Other^a^	Race not reported	Total
With race data	1501 (45.2)	1055 (31.7)	375 (11.3)	393 (11.8)	3324 (100)
Reported ≥1 unmet social need	540 (36.0)	115 (10.9)	108 (28.8)	144 (36.6)	907 (27.3)
Reported ≥2 unmet social needs	342 (22.8)	66 (6.3)	78 (20.8)	104 (26.5)	590 (17.8)
Reported ≥3 unmet social needs	204 (13.7)	33 (3.1)	43 (11.5)	204 (51.9)	345 (10.4)
Difficulty paying for utilities	318 (21.2)	54 (5.1)	73 (19.5)	93 (23.7)	538 (16.2)
Difficulty paying for food or obtaining food due to isolation or quarantine	321 (21.4)	49 (4.6)	61 (16.3)	91 (23.2)	522 (15.7)
Difficulty paying for housing	284 (18.9)	64 (6.1)	72 (19.2)	86 (21.9)	506 (15.2)
Difficulty paying for medications	140 (9.3)	29 (2.7)	33 (8.8)	34 (8.7)	236 (7.1)
Difficulty obtaining transportation to see a health care provider	123 (8.2)	23 (2.2)	22 (5.9)	39 (9.9)	207 (6.2)
In danger of being evicted	38 (2.5)	10 (0.9)	11 (2.9)	14 (3.6)	73 (2.2)

^a^ Other category includes Asian, American Indian or Alaskan Native, Native Hawaiian or Pacific Islander, or reported by participant as unspecified “other.”

Black participants were more likely than participants of other self-reported races to experience unmet social needs. Among 1501 Black participants with data on unmet social needs, 540 individuals (36.0%) reported at least 1 unmet social need, vs 115 of 1055 White participants (10.9%) and 108 of 375 participants with other races (28.8%) (Table 2). Compared with Black participants, White participants had significantly lower odds of reporting unmet social needs (OR, 0.20; 95% CI, 0.16-0.25).

Additional qualitative data gathered from team communications demonstrated the often-pressing concerns facing patients and their contacts. For example, one volunteer said, “[Contact] is refusing to isolate because when they were sick…with COVID, they weren't paid for 3 weeks and couldn’t make bills. They work at a nursing home…they can't miss work and will lose their house.” When faced with such fundamental threats, cases and contacts often had to make difficult decisions that conflicted with isolation and quarantine guidance (eg, going to work while infectious).

Throughout the program, our team met to discuss quality improvement and operational challenges. We noted and responded to shortcomings in initial program design, particularly as they related to coordination of efforts between our health system–based team and PDPH. For example, we developed systems for rapidly identifying and referring to the appropriate LHDs cases that had a high likelihood of onward transmission or potential for generating outbreaks in high-risk populations. Such HERCs were directed to the immediate attention of an outbreak investigations professional at PDPH, as described in this April 18, 2020, memo from our team to University of Penn and PDPH leadership: “Our team will send PDPH’s report form for severe/high-risk setting cases via secure email…HERCs may be identified during CI interviews, in which case they will be reported to PDPH using the same form/email…we will have a dedicated Reporting Team that will exclusively be responsible for reporting HERCs to PDPH.”

We also grappled with a second example related to interjurisdictional CI and CT challenges commonly faced by LHDs. While PDPH is responsible for the health of only Philadelphia County residents and therefore conducted CI and CT only for individuals residing within county limits, UPHS has a broader catchment area. Thus, individuals tested at UPHS sites lived in many municipalities, some of which did not have health department capacity for CI and CT, resulting in unanswered emails, as shown in this October 2021 email: “...in our contact tracing, [we] identified a wedding that was held in [redacted]. It’s unlikely that all of the wedding guests are from [redacted], but I wanted to see if you have any information that might be relevant to our investigation (while protecting confidentiality).”

To foster communication with LHDs and streamline data sharing, we attempted multiple strategies, including the purchase of a site license for the CI and CT software used by PDPH and many (but not all) neighboring health departments. However, the private company that developed this software responded that it was unable to fulfill our request, because it, too, was understaffed to meet pandemic demands for its services (recorded in an email dated August 4, 2020).

## Discussion

In this case series, we described the design, implementation, and accomplishments of a 15-month volunteer-staffed CI and CT program run within a large academic health system in collaboration with the LHD. At multiple points throughout the pandemic, caseloads have outstripped the capacity of health departments to conduct CI and CT. In response, many institutions established stopgap CI and CT teams to augment health department capacity.^7,8,9,10,16,17,18,19^ This study’s findings suggest the acceptability of a health system conducting COVID-19 CI and CT within its patient population, as well as the role that CI and CT volunteers may play in addressing a range of unmet needs.

During the COVID-19 pandemic, a prevailing narrative emerged that individuals in the US were unwilling to participate in CI or CT.^20^ In contrast, we found that most cases and contacts responded to our calls, similar to reports from a large study^21^ of health departments, which reported an interview rate of 59%. It is well documented that contact tracing is associated with prevention of transmission of infectious diseases like Ebola and tuberculosis,^22,23^ as well as COVID-19 when implemented with other mitigation measures.^24,25,26,27,28^ There is also good evidence that CI and CT are highly cost-effective, but the US has not yet built the necessary infrastructure or invested in optimizing the process or its implementation.^29,30^

A key question highlighted by our experience is how to achieve optimal staffing for CI and CT efforts given the highly fragmented, localized approach to public health in the US. In this program, most staff effort was donated by volunteers, with philanthropic and in-kind university support for program management. In the context of a waxing and waning pandemic and limited resources, it is neither financially nor operationally reasonable for LHDs to maintain staffing for peak cases; however, without surge staffing readily available, jurisdictions would need to ration CI and CT when case numbers increase. A potential solution is to establish state, regional, or national CI and CT corps as a shared resource to provide support remotely, integrating services across geographic lines and responding promptly to stamp out incipient outbreaks. Having sufficient capacity at a regional and national level may be the only way to provide a buffer against swings in local needs, much like a national PPE stockpile.^31^

This study’s results also suggest that CI and CT teams are important conduits of health information, filling critical gaps in health care and public health services. We provided verbal and written guidance, and sometimes this was the only guidance participants received regarding their diagnosis or exposure. At the point of testing for COVID-19, almost 25% of participants received no information about isolation or infection control. Most participants who received any information at the point of testing received verbal instructions, which are generally considered ineffective for conveying health information.^32,33^ This communication gap is especially concerning because 1 in 4 US adults do not have a primary care physician.^34^ In the absence of a primary care physician, CI and CT programs address fundamental questions, such as when to safely return to work and when to seek emergency care. Additionally, CI and CT programs provide letters to employers to excuse work absence. Thus, the dissolution of CI and CT programs is especially harmful to people without primary care access.

Finally, this study suggests that CI and CT programs may facilitate quarantine and isolation, especially for low-resource populations, which is essential to an equity-focused pandemic response. Nearly 30% of our participants reported at least 1 unmet social need, which CI and CT volunteers addressed through referral to the SNRT, which linked people to services like food delivery. Furthermore, more than half of our participants were Black, and Black participants were more likely than White participants to report unmet social needs that interfered with isolation or quarantine. Systemic racism has created these inequities, which can be exacerbated by disinvestment in prevention strategies like CI and CT.^35,36,37^

### Limitations

This work had several limitations, chiefly that the data were collected for emergency pandemic response and not for research. Additionally, REDCap is typically used for research data collection rather than for case investigation and documentation of close contacts. A system designed for CI and CT should automate routine tasks, such as notifying relevant health departments of close contacts and sharing written guidance. These tasks were instead conducted manually. A challenge that must be remedied is the lack of a universal data-sharing platform for conducting CI and CT that enables interinstitution and crossjurisdiction communication and provides sufficient customization to address variation in policies and procedures.

## Conclusions

This case series demonstrated the role of a health system-based CI and CT initiative in coaching patients through public health guidance and linking them to vital social resources. CI and CT programs across the country, including our own, were implemented rapidly to address a global catastrophe and were largely disbanded midpandemic, thus eliminating a mitigation strategy and a potential health support, especially for high-risk communities. In the absence of robust, adequately resourced public health infrastructure, health systems should consider their role in the nonclinical components of epidemic control like social, informational, and material support for the current and future pandemics.

## Notes

Supplement. **eFigure 1.** Case Investigation and Contact Tracing Program Timeline **eFigure 2.** COVID-19 Volunteer Opportunities Interest Form **eFigure 3.** Follow-up Resources for Cases and Contacts **eMethods.** Description of Team Roles and Responsibilities
