# Supplementary material for: Design, Implementation, and Outcomes of a Volunteer-Staffed Case Investigation and Contact Tracing Initiative at an Urban Academic Medical Center
Source: JAMA Netw Open. 2022 Sep 23;5(9):e2232110. doi: 10.1001/jamanetworkopen.2022.32110 (PMC9508658; doi:10.1001/jamanetworkopen.2022.32110)
Supplement: Supplement. — eFigure 1. Case Investigation and Contact Tracing Program Timeline eFigure 2. COVID-19 Volunteer Opportunities Interest Form eFigure 3. Follow-up Resources for Cases and Contacts eMethods. Description of Team Roles and Responsibilities [file jamanetwopen-e2232110-s001.pdf]

Supplemental Online Content

Feuerstein-Simon R, Strelau KM, Naseer N, et al. Design, implementation, and outcomes of a volunteer-staffed case investigation and contact tracing initiative at an urban academic medical center. *JAMA Netw Open*. 2022;5(9):e2232110. doi:10.1001/jamanetworkopen.2022.32110

eFigure 1. Case Investigation and Contact Tracing Program Timeline
eFigure 2. COVID-19 Volunteer Opportunities Interest Form
eFigure 3. Follow-up Resources for Cases and Contacts
eMethods. Description of Team Roles and Responsibilities

This supplemental material has been provided by the authors to give readers additional information about their work.

© 2022 Feuerstein-Simon R et al. *JAMA Network Open.*

eFigure 1. Case Investigation and Contact Tracing Program Timeline

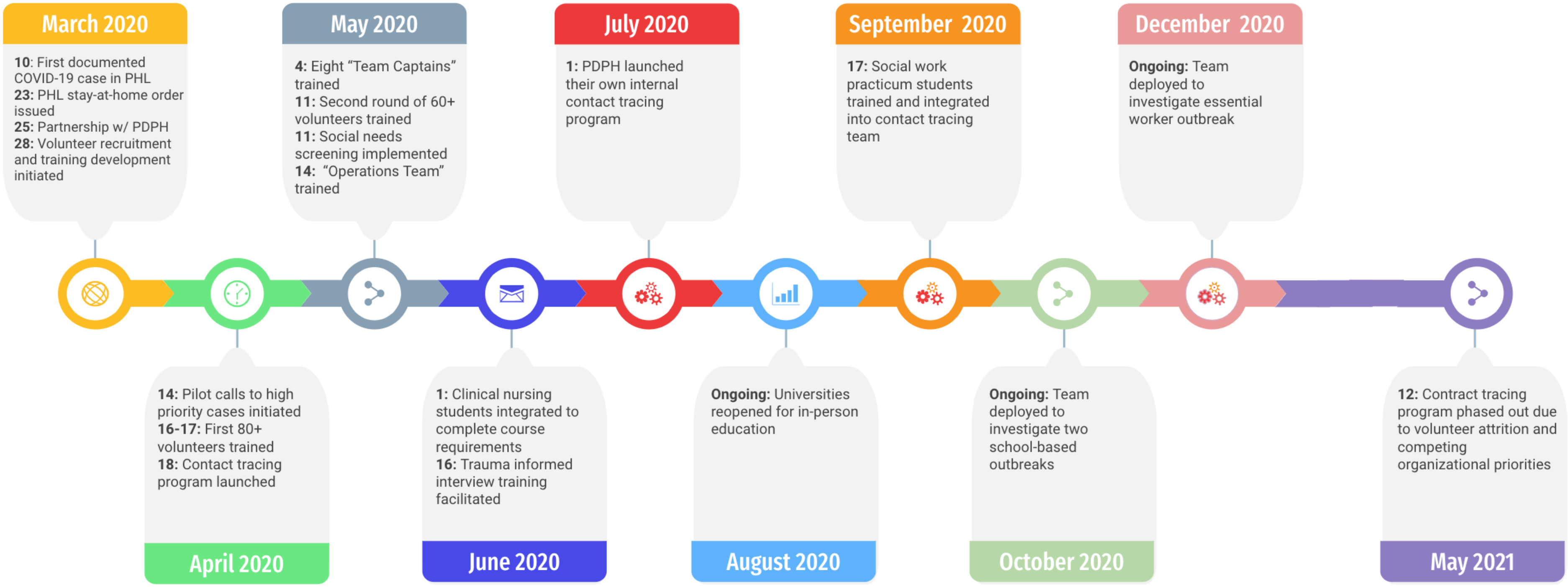

© 2022 Feuerstein-Simon R et al. *JAMA Network Open.*

eFigure 2. COVID-19 Volunteer Opportunities Interest Form

Volunteer form: Philadelphia Department of Public Health COVID19

* Required

1. Email *

   ____

2. Last Name *

   ____

3. First Name *

   ____

4. Are you currently a Penn student? *

   Mark only one oval.

   ( ) Yes
   ( ) No

5. If you are a student, please tell us what program (select more than one if you are a dual degree student):

   Check all that apply.

   [ ] Public Health
   [ ] Nursing
   [ ] Social Work
   [ ] Medicine
   [ ] Other: ______________________

6. If you are not currently a Penn student, where do you work and what is your job title?

   ____

© 2022 Feuerstein-Simon R et al. *JAMA Network Open.*

7. Which of the following volunteer positions would you be willing to fill? (Please select ALL that you would be willing to perform) *

Check all that apply.

- [ ] Contact tracing -- This role involves contacting COVID-19+ individuals by phone with a commitment of approximately 2-4 hours per day for 10 weeks.
- [ ] Monitoring -- This role involves phone, text, and email outreach to persons under investigation (PUI) and COVID-19+ individuals with a commitment of 2-4 hours per day for 10 weeks.
- [ ] Social media -- This role involves scanning social media platforms (Twitter, Facebook) to identify rumors/misinformation and/or ensure accurate information. This has a flexible time commitment.
- [ ] Written material adaptation -- This role requires volunteers with strong communication skills who can screen and edit fact sheets/ documents for low-literacy audiences and translate to other languages. This time commitment is flexible.

8. These volunteer positions will require a set weekly time commitment (varying by role). Please describe any fixed competing obligations in your schedule (e.g., weekly classes, family obligations): *

9. Approximately how many hours PER WEEK would you be available to commit to this volunteer position? *

10. Please list any languages (other than English) that you can speak, read, or understand:

11. Please describe three strengths you will bring to this volunteer position: *

© 2022 Feuerstein-Simon R et al. *JAMA Network Open.*

12. The outbreak of coronavirus disease 2019 (COVID-19) is undoubtedly stressful for * all of us. Fear and anxiety about the disease, well-being of family and friends, strained financial resources, and overall changes in day-to-day life can be overwhelming and cause strong emotions in adults and children. Imagine you have a 'distress thermometer' to measure your feelings according to the following scale (0-10).

Mark only one oval.

- 0: Relaxed and concentrating well.
- 1
- 2
- 3: Mildly upset. Worried, bothered to the point that you notice it.
- 4
- 5: Moderate anxiety/distress, uncomfortable but can continue to perform.
- 6
- 7: Quite anxious, distressed. Interfering with functioning.
- 8
- 9
- 10: Highest distress/fear/anxiety/discomfort that you have ever felt.

© 2022 Feuerstein-Simon R et al. *JAMA Network Open.*

eFigure 3. Follow-up Resources for Cases and Contacts

Coronavirus Disease 2019 (COVID-19) Fact Sheet (Cases)

Penn Medicine: Self-schedule an appointment via myPennMedicine.org or call the Access Center (215-615-2222) and say it's related to Covid-19

Philadelphia Coronavirus Helpline: (800) 722-7112

Philadelphia Department of Health: text COVIDPHL to 888-777 or visit phila.gov/COVID-19

What are the signs and symptoms of COVID-19?

How is COVID-19 transmitted?

Where can a person get tested for COVID-19?

Now that I have tested positive, how long will I be able to infect other people?

How can I protect other people from getting infected?

Where can I get help with social needs?

How can I get groceries while in physical isolation?

How can I cope with the stress caused by the COVID-19 pandemic?

How can I access Protection from Abuse Orders and other domestic/intimate partner violent services?

How can I access unemployment resources?

Where can I report unsafe practices in my workplace?

Once I have recovered from COVID-19, where can I donate plasma?

What resources are available for my family?

Where can I find more information about COVID-19?

© 2022 Feuerstein-Simon R et al. *JAMA Network Open.*

What are the signs and symptoms of COVID-19?

In most people, COVID-19 presents as a typical upper respiratory infection. Symptoms appear between 2-14 days after exposure to someone else with the infection. Symptoms most often reported include:

- Fever
- Cough
- Shortness of breath or difficulty breathing
- Chills
- Repeated shaking with chills
- Muscle pain
- Headache
- Sore throat
- New loss of taste or smell

Severe symptoms that require **immediate medical attention** include:

- Extremely difficult breathing (not being able to speak without gasping for air)
- Bluish lips or face
- Persistent pain or pressure in the chest
- Severe persistent dizziness or lightheadedness
- New confusion, or inability to arouse
- New seizure or seizures that will not stop

Based on the currently available information, people most vulnerable to serious complications from COVID-19 include:

- Those with underlying health problems such as diabetes, lung disease, or heart disease
- Those who are immunocompromised
- Those who are older than 65 years

How is COVID-19 transmitted?

- People can become sick with COVID-19 after being infected with the virus called SARS-CoV-2.
- The virus is transmitted through respiratory droplets, mainly through person-to-person interaction. When an infected person coughs, sneezes, or exhales, tiny droplets containing the virus are released. People can become infected by breathing in these droplets.
- The virus can also be transmitted by touching inanimate objects called fomites. The respiratory droplets can land on surfaces or objects. The available information estimates that the virus can survive on surfaces from a few hours up to several days. People can become infected by touching these surfaces and then touching their mouth, nose, or eyes.
- Both people with symptoms (symptomatic) and people without symptoms (asymptomatic) can spread the disease.
- Based on the available information, it is unknown how large an exposure to the virus results in an infection.
- Based on the available information, it is unknown whether or not a person can become infected with SARS-CoV-2 more than one time.

Where can a person get tested for COVID-19?

© 2022 Feuerstein-Simon R et al. *JAMA Network Open.*

- Penn Medicine has several coronavirus testing sites. The hours and policies may differ by location. Call the Penn Medicine Coronavirus Hotline for up-to-date details.
    - For more information about testing locations, see COVID-19 Drive-Thru Testing Sites.
    - At Penn Medicine, a person can get tested without having a primary care provider or health insurance.
- For more information about COVID-19 testing services outside of Penn Medicine, see COVID-19 Testing.

Now that I have tested positive, how long will I be able to infect other people?

- It is likely that you were infectious (able to infect others) before you experienced symptoms.
- The time between getting infected with SARS-CoV-2 and the beginning of symptoms is called the incubation period. On average, the incubation period is 3-5 days with a range of 2-14 days.
    - Both infected people with symptoms (symptomatic) and people without symptoms (asymptomatic) are able to infect other people.
- Based on the available information, the infectious period (the time when you are contagious and able to infect others) is not yet known.

How can I protect other people from getting infected?

Physically isolate yourself

- Do not leave your home and restrict visitors to your home.
- Stay in one room in the home away from other people.
    - For more information on shared living spaces, see Households living in close quarters and Living in shared housing.
- If possible, use a separate bathroom.
- Avoid sharing personal items, such as dishes, towels, and bedding.
- If you must leave your home to seek immediate medical attention, notify the 911 operator about COVID-19, if possible.
- You should remain in isolation until: 1) you have had no fever for at least 24 hours without the use of medicine that reduces fever; 2) other symptoms have improved (for example, your cough and shortness of breath have improved); and 3) at least 10 days have passed since your symptoms first appeared.
    - Note that instructions are different for health care workers and for those who are residents of long-term care facilities, shelters, or other congregate settings.
    - For more information, see Criteria to discontinue quarantine or isolation.
    - Penn Medicine does not re-test patients unless there is a significant worsening in clinical condition, such as a hospitalization. However, other community testing sites may advise differently.

Wash your hands

- Regularly wash your hands with soap and water for at least 20 seconds.
- If soap and water are not readily available, use a hand sanitizer that contains at least 60% alcohol. Cover all surfaces of your hands and rub them together until they feel dry.
- Avoid touching your eyes, nose, and mouth.

© 2022 Feuerstein-Simon R et al. *JAMA Network Open.*

Cover your coughs and sneezes

- Cough and sneeze into a tissue or your upper sleeve.
- Throw the used tissue into a trash can with a plastic bag in it.
- Immediately wash your hands.

Wear a cloth face covering

- If you must be around other people, wear a cloth face covering.
    - Medical masks (surgical and N95) should be reserved for healthcare workers and first responders.
- The cloth face covering should cover your nose and mouth.
- Wash your cloth face covering after each use.
- For more information about making and wearing a cloth face covering, see Use of cloth face coverings to help slow the spread of COVID-19.

Disinfect your home

- You should disinfect the room in which you are physically isolating. Another person in the house should disinfect the common areas.
    - If you cannot clean and disinfect your room, the other person should wear a mask and disposable gloves to clean your room on an as-needed basis.
- First, clean to remove germs and dirt.
    - Use the household cleaner that you typically use and follow the directions on the label.
- Second, disinfect surfaces.
    - Most disinfectants are effective against the coronavirus that causes COVID-19. For more information, see Disinfectants for use against SARS-CoV-2.
    - Diluted household bleach solutions and alcohol solutions with at least 70% alcohol can also be used as disinfectants.
- Disinfect commonly touched surfaces, such as light switches, doorknobs, railings, chairs, tables, handles, faucets, and telephones every day.

Wash your laundry thoroughly

- Wear disposable gloves when handling your dirty laundry. After removing the gloves, throw them in the trash and wash your hands with soap and water.
- Laundry can be washed in a standard washing machine with detergent and hot water (at least 140°F). It is not necessary to separate soiled linen and laundry from infected people from that of other household members.

Where can I get help with social needs?

Penn Medicine has a Social Needs Response Team of licensed social workers and trained professionals to assist you with your needs. If you are experiencing distress, have safety concerns, or need assistance with resources such as food, housing, transportation, medications, loss of health insurance, or unemployment benefits, contact the **COVID-19 Social Needs Response Team** at 267-785-2019 Monday-Friday, 8am-8pm.

How can I get groceries while in physical isolation?

© 2022 Feuerstein-Simon R et al. *JAMA Network Open.*

- Have a housemate who has not be in contact with you pick up your groceries.
- If you live alone, get your groceries delivered through mobile applications and websites.
    - Get your medications delivered as well. Many pharmacies will deliver your prescriptions at no additional cost. Call your pharmacy to see if they are delivering medications.
- If possible, stock up on non-perishable food (canned foods, dried beans, pasta) to minimize trips to the store.
- If you need help getting free and nutritious food, the city has established food sites (any resident can pick up free food for their household) and student meal sites (any child can pick up free meals). Locations for food pantries can be found by:
    - Phone: Call 311 for help finding a food pantry. Make sure to call the pantry in advance to confirm their hours.
    - Text: Text your zip code to 1-800-548-6479 to receive a list of food pantries near you.
    - Online: The City of Philadelphia website for COVID-19 resources
    - Call food banks directly to see if they deliver.

How can I cope with the stress caused by the COVID-19 pandemic?

The pandemic of COVID-19 may be stressful for people. Fear and anxiety about the disease can be overwhelming and cause strong emotions in adults and children. Coping with stress will make you, the people you care about, and your community stronger. Stress during this time can include:

- Fear and worry about your own health and the health of your loved ones
- Changes in sleep or eating patterns
- Difficulty sleeping or concentrating
- Worsening of chronic health problems
- Worsening of mental health conditions
- Increased use of alcohol, tobacco, or other drugs

Understand the risks to yourself and other people

Take care of your mental health

- Limit exposure to media discussing the COVID-19 pandemic.
- Connect with loved ones through virtual platforms.
- Promote physical well-being by stretching and exercising.
- Practice mediation and take deep breaths.
- Try to get the recommended eight hours of sleep every night.
- If you have a preexisting mental health condition, you should continue your treatment and monitor new or worsening symptoms.

Reach out for help

- Call your healthcare provider if stress gets in the way of your daily activities for several days in a row.
- If you feel lonely, call the Philadelphia Warm-line at 1-855-507-9276 to talk to a trained peer specialist.

© 2022 Feuerstein-Simon R et al. *JAMA Network Open.*

- Visit the Disaster Distress Helpline, call 1-800-985-5990, or text TalkWithUs to 66746.
- For information about substance abuse and mental health services, see (SAMHSA) Disaster Preparedness.

If you, or someone you care about, are feeling overwhelmed with emotions like sadness, depression, or anxiety, or feel like you want to harm yourself or others

- Call 911.
- Call the suicide and crisis intervention hotline at 215-686-4420.
- Text the Crisis Text Line at 741741 to connect with a trained volunteer Crisis Counselor for help.

How can I access Protection from Abuse Orders and other domestic/intimate partner violent services?

- Call the Women Against Abuse Legal Center at (215) 686-7082 and leave a message with a safe number for an advocate to call you back.
- Call the Philadelphia Domestic Violence Hotline at (866) 723-3014.
- Visit the National Domestic Violence Hotline or call 1-800-799-7233 and TTY 1-800-787-3224.
- For more information about protection from abuse orders and other domestic violence resources, visit the Women's Law Project.

How can I access unemployment resources?

- You may be eligible for unemployment compensation from the Commonwealth of Pennsylvania if:
  - Your employer temporarily closes or goes out of business because of COVID-19.
  - Your employer reduces your hours because of COVID-19.
  - You have been told not to work because your employer feels you might get or spread COVID-19.
  - You have been told to quarantine or self-isolate, or you live or work in a county under government-recommended mitigation efforts.
- The Commonwealth is waiving wait time and work requirements for applicants. Learn how to apply online for unemployment compensation. If you need assistance completing the application, visit the Philadelphia Legal Assistance resource page or call (215) 999-6910.
- For more information, see Information for workers.

Where can I report unsafe practices in my workplace?

If you are concerned about unsafe COVID-19 related practices or policies in your workplace, you can report the issue.

- If you are a city employee:
  - Call City of Philadelphia 311 hotline.
  - Visit the Philly 311 webpage.
  - Call the business service hotline at (215)-683-2100.
- If you are a non-city employee, you can submit complaints using the PA State Department of Health form. For more information, see COVID-19 Complaint Form.

Once I have recovered from COVID-19, where can I donate plasma?

- Once you recover from COVID-19, your plasma (liquid component of the blood) will contain antibodies against the SARS-CoV-2 that may be helpful to people who have not yet recovered from the disease.
- Requirements for donation may be different depending on the location.
    - For more information about donating at Penn Medicine, see Plasmapheresis Donor Registry for Patients Recovered from Confirmed COVID-19.
    - For more information about donating at the American Red Cross, see Plasma Donations from Recovered COVID-19 Patients.
    - For more information about donating at Jefferson Health, see Coronavirus (COVID-19) Plasma Donation and Registry.

What resources are available for my family?

- K-6 children with parents working outside of the home and do not have access to childcare may be eligible to register to attend the Digital Learning Access Centers.
    - Centers open September 8th, Monday-Friday 8:00AM-4:30PM
    - For more information, see Access Centers.
- Families with school-age children not eligible for Access Center services, and with no or inadequate internet access or technology support, may be eligible to receive internet services and a laptop computer for education use from PHLConnectEd.
    - For more information, see PHLConnectED.
- Resources for families and early learning providers during COVID-19
- Free pregnancy, baby, and toddler support during COVID-19
- Health and wellness tips for parents of young children during COVID-19
- Resources and tips for digital learning while Philly schools are closed
- Staying healthy and seeking non-COVID-19 medical care

Where can I find more information about COVID-19?

- Philadelphia Department of Public Health
- Centers for Disease Control and Prevention
- Penn Medicine

© 2022 Feuerstein-Simon R et al. *JAMA Network Open.*

Coronavirus Disease 2019 (COVID-19) Fact Sheet (Contacts)

Philadelphia Coronavirus Helpline: (800) 722-7112
Philadelphia Department of Health: text COVIDPHL to 888-777 or visit phila.gov/COVID-19

What are the signs and symptoms of COVID-19?

How is COVID-19 transmitted?

Where can I get tested for COVID-19?

If I test positive, how long will I be able to infect other people?

If I test positive, how can I protect other people from getting infected?

Where can I get help with social needs?

How can I get groceries if I have to be in physical isolation?

If I test negative, how can I protect myself and other people from getting sick?

How can I cope with the stress caused by the COVID-19 pandemic?

How can I access Protection from Abuse Orders and other domestic/intimate partner violent services?

How can I access unemployment resources?

Where can I report unsafe practices in my workplace?

If I get infected and recover from COVID-19, where can I donate plasma?

What resources are available for my family?

Where can I find more information about COVID-19?

What are the signs and symptoms of COVID-19?

In most people, COVID-19 presents as a typical upper respiratory infection. Symptoms appear between 2-14 days after exposure to someone else with the infection. Symptoms most often reported include:

- Fever
- Cough
- Shortness of breath or difficulty breathing
- Chills
- Repeated shaking with chills
- Muscle pain
- Headache
- Sore throat

© 2022 Feuerstein-Simon R et al. *JAMA Network Open.*

- New loss of taste or smell

Severe symptoms that require **immediate medical attention** include:
- Extremely difficult breathing (not being able to speak without gasping for air)
- Bluish lips or face
- Persistent pain or pressure in the chest
- Severe persistent dizziness or lightheadedness
- New confusion, or inability to arouse
- New seizure or seizures that will not stop

Based on the currently available information, people most vulnerable to serious complications from COVID-19 include:
- Those with underlying health problems such as diabetes, lung disease, or heart disease
- Those who are immunocompromised
- Those who are older than 65 years

How is COVID-19 transmitted?
- People can become sick with COVID-19 after being infected with the virus called SARS-CoV-2.
- The virus is transmitted through respiratory droplets, mainly through person-to-person interaction. When an infected person coughs, sneezes, or exhales, tiny droplets containing the virus are released. People can become infected by breathing in these droplets.
- The virus can also be transmitted by touching inanimate objects called fomites. The respiratory droplets can land on surfaces or objects. The available information estimates that the virus can survive on surfaces from a few hours up to several days. People can become infected by touching these surfaces and then touching their mouth, nose, or eyes.
- Both people with symptoms (symptomatic) and people without symptoms (asymptomatic) can spread the disease.
- Based on the available information, it is unknown how large an exposure to the virus results in an infection.
- Based on the available information, it is unknown whether or not a person can become infected with SARS-CoV-2 more than one time.

Where can I get tested for COVID-19?
- Penn Medicine has several coronavirus testing sites. The hours and policies may differ by location. Call the Penn Medicine Coronavirus Hotline for up-to-date details.
  - For more information about testing locations, see COVID-19 Drive-Thru Testing Sites.
  - At Penn Medicine, a person can get tested without having a primary care provider or health insurance.
- For more information about COVID-19 testing services outside of Penn Medicine, see COVID-19 Testing.

If I test positive, how long will I be able to infect other people?
- It is likely that you were infectious (able to infect others) before you experienced symptoms.

© 2022 Feuerstein-Simon R et al. *JAMA Network Open.*

- The time between getting infected with SARS-CoV-2 and the beginning of symptoms is called the incubation period. On average, the incubation period is 3-5 days with a range of 2-14 days.
    - Both infected people with symptoms (symptomatic) and people without symptoms (asymptomatic) are able to infect other people.
- Based on the available information, the infectious period (the time when you are contagious and able to infect others) is not yet known.

If I test positive, how can I protect other people from getting infected?

Physically isolate yourself

- Do not leave your home, and restrict visitors to your home.
- Stay in one room in the home away from other people.
    - For more information on shared living spaces, see Households living in close quarters and Living in shared housing.
- If possible, use a separate bathroom.
- Avoid sharing personal items, such as dishes, towels, and bedding.
- If you must leave your home to seek immediate medical attention, notify the 911 operator about COVID-19, if possible.
- You should remain in isolation until: 1) you have had no fever for at least 24 hours without the use of medicine that reduces fever; 2) other symptoms have improved (for example, your cough and shortness of breath have improved); and 3) at least 10 days have passed since your symptoms first appeared.
    - Note that instructions are different for health care workers and for those who are residents of long-term care facilities, shelters, or other congregate settings.
    - For more information, see Criteria to discontinue quarantine or isolation.
    - Penn Medicine does not re-test patients unless there is a significant worsening in clinical condition, such as a hospitalization. However, other community testing sites may advise differently.

Wash your hands

- Regularly wash your hands with soap and water for at least 20 seconds.
- If soap and water are not readily available, use a hand sanitizer that contains at least 60% alcohol. Cover all surfaces of your hands and rub them together until they feel dry.
- Avoid touching your eyes, nose, and mouth.

Cover your coughs and sneezes

- Cough and sneeze into a tissue or your upper sleeve.
- Throw the used tissue into a trash can with a plastic bag in it.
- Immediately wash your hands.

Wear a cloth face covering

- If you must be around other people, wear a cloth face covering.
    - Medical masks (surgical and N95) should be reserved for healthcare workers and first responders.
- The cloth face covering should cover your nose and mouth.
- Wash your cloth face covering after each use.

© 2022 Feuerstein-Simon R et al. *JAMA Network Open.*

- For more information about making and wearing a cloth face covering, see Use of cloth face coverings to help slow the spread of COVID-19.

Disinfect your home

- You should disinfect the room in which you are physically isolating. Another person in the house should disinfect the common areas.
    - If you cannot clean and disinfect your room, the other person should wear a mask and disposable gloves to clean your room on an as-needed basis.
- First, clean to remove germs and dirt.
    - Use the household cleaner that you typically use and follow the directions on the label.
- Second, disinfect surfaces.
    - Most disinfectants are effective against the coronavirus that causes COVID-19. For more information, see Disinfectants for use against SARS-CoV-2.
    - Diluted household bleach solutions and alcohol solutions with at least 70% alcohol can also be used as disinfectants.
- Disinfect commonly touched surfaces, such as light switches, doorknobs, railings, chairs, tables, handles, faucets, and telephones every day.

Wash your laundry thoroughly

- Wear disposable gloves when handling your dirty laundry. After removing the gloves, throw them in the trash and wash your hands with soap and water.
- Laundry can be washed in a standard washing machine with detergent and hot water (at least 140°F). It is not necessary to separate soiled linen and laundry from infected people from that of other household members.

Where can I get help with social needs?

Penn Medicine has a Social Needs Response Team of licensed social workers and trained professionals to assist you with your needs. If you are experiencing distress, have safety concerns, or need assistance with resources such as food, housing, transportation, medications, loss of health insurance, or unemployment benefits, contact the **COVID-19 Social Needs Response Team** at 267-785-2019 Monday-Friday, 8am-8pm.

How can I get groceries if I have to be in physical isolation?

- Have a housemate who has not be in contact with you pick up your groceries.
- If you live alone, get your groceries delivered through mobile applications and websites.
    - Get your medications delivered as well. Many pharmacies will deliver your prescriptions at no additional cost. Call your pharmacy to see if they are delivering medications.
- If possible, stock up on non-perishable food (canned foods, dried beans, pasta) to minimize trips to the store.
- If you need help getting free and nutritious food, the city has established food sites (any resident can pick up free food for their household) and student meal sites (any child can pick up free meals). Locations for food pantries can be found by:
    - Phone: Call 311 for help finding a food pantry. Make sure to call the pantry in advance to confirm their hours.

© 2022 Feuerstein-Simon R et al. *JAMA Network Open.*

    - Text: Text your zip code to 1-800-548-6479 to receive a list of food pantries near you.
    - Online: The City of Philadelphia website for COVID-19 resources
    - Call food banks directly to see if they deliver.

If I test negative, how can I protect myself and other people from getting sick?

Self-quarantine

- The test for SARS-CoV-2 does not detect the virus 100% of the time. Even if you tested negative, it is possible that you are infected. This is called a "false negative" result.
- Stay in your home for 14 days after coming into contact with someone who has COVID-19.
    - If you live with someone who has COVID-19, stay in quarantine for 14 days after the person ends home isolation per the criteria listed above for physical isolation. (Date the person with COVID-19 ends home isolation + 14 days= end of your quarantine)
- Monitor your symptoms.

Avoid close contact with other people

- Stay at least six feet away from other people.
- Avoid crowded places and do not gather in groups of people.
- Reduce the number of times you visit the grocery store and other essential businesses.
- If possible, work from home.

Practice hygiene and mask behaviors described as if you tested positive

How can I cope with the stress caused by the COVID-19 pandemic?

The pandemic of COVID-19 may be stressful for people. Fear and anxiety about the disease can be overwhelming and cause strong emotions in adults and children. Coping with stress will make you, the people you care about, and your community stronger. Stress during this time can include:

- Fear and worry about your own health and the health of your loved ones
- Changes in sleep or eating patterns
- Difficulty sleeping or concentrating
- Worsening of chronic health problems
- Worsening of mental health conditions
- Increased use of alcohol, tobacco, or other drugs

Understand the risks to yourself and other people

Take care of your mental health

- Limit exposure to media discussing the COVID-19 pandemic.
- Connect with loved ones through virtual platforms.
- Promote physical well-being by stretching and exercising.
- Practice mediation and take deep breaths.
- Try to get the recommended eight hours of sleep every night.

© 2022 Feuerstein-Simon R et al. *JAMA Network Open.*

- If you have a preexisting mental health condition, you should continue your treatment and monitor new or worsening symptoms.

Reach out for help

- Call your healthcare provider if stress gets in the way of your daily activities for several days in a row.
- If you feel lonely, call the Philadelphia Warm-line at 1-855-507-9276 to talk to a trained peer specialist.
- Visit the Disaster Distress Helpline, call 1-800-985-5990, or text TalkWithUs to 66746.
- For information about substance abuse and mental health services, see (SAMHSA) Disaster Preparedness.

If you, or someone you care about, are feeling overwhelmed with emotions like sadness, depression, or anxiety, or feel like you want to harm yourself or others

- Call 911.
- Call the suicide and crisis intervention hotline at 215-686-4420.
- Text the Crisis Text Line at 741741 to connect with a trained volunteer Crisis Counselor for help.

How can I access Protection from Abuse Orders and other domestic/intimate partner violent services?

- Call the Women Against Abuse Legal Center at (215) 686-7082 and leave a message with a safe number for an advocate to call you back.
- Call the Philadelphia Domestic Violence Hotline at (866) 723-3014.
- Visit the National Domestic Violence Hotline or call 1-800-799-7233 and TTY 1-800-787-3224.
- For more information about protection from abuse orders and other domestic violence resources, visit the Women's Law Project.

How can I access unemployment resources?

- You may be eligible for unemployment compensation from the Commonwealth of Pennsylvania if:
  - Your employer temporarily closes or goes out of business because of COVID-19.
  - Your employer reduces your hours because of COVID-19.
  - You have been told not to work because your employer feels you might get or spread COVID-19.
  - You have been told to quarantine or self-isolate, or you live or work in a county under government-recommended mitigation efforts.
- The Commonwealth is waiving wait time and work requirements for applicants. Learn how to apply online for unemployment compensation. If you need assistance completing the application, visit the Philadelphia Legal Assistance resource page or call (215) 999-6910.
- For more information, see Information for workers.

Where can I report unsafe practices in my workplace?

If you are concerned about unsafe COVID-19 related practices or policies in your workplace, you can report the issue.

- If you are a city employee:
    - Call City of Philadelphia 311 hotline.
    - Visit the Philly 311 webpage.
    - Call the business service hotline at (215)-683-2100.
- If you are a non-city employee, you can submit complaints using the PA State Department of Health form. For more information, see COVID-19 Complaint Form.

If I get infected and recover from COVID-19, where can I donate plasma?

- Once you recover from COVID-19, your plasma (liquid component of the blood) will contain antibodies against the SARS-CoV-2 that may be helpful to people who have not yet recovered from the disease.
- Requirements for donation may be different depending on the location.
    - For more information about donating at Penn Medicine, see Plasmapheresis Donor Registry for Patients Recovered from Confirmed COVID-19.
    - For more information about donating at the American Red Cross, see Plasma Donations from Recovered COVID-19 Patients.
    - For more information about donating at Jefferson Health, see Coronavirus (COVID-19) Plasma Donation and Registry.

What resources are available for my family?

- K-6 children with parents working outside of the home and do not have access to childcare may be eligible to register to attend the Digital Learning Access Centers.
    - Centers open September 8th, Monday-Friday 8:00AM-4:30PM
    - For more information, see Access Centers.
- Families with school-age children not eligible for Access Center services, and with no or inadequate internet access or technology support, may be eligible to receive internet services and a laptop computer for education use from PHLConnectEd.
    - For more information, see PHLConnectED.
- Resources for families and early learning providers during COVID-19
- Free pregnancy, baby, and toddler support during COVID-19
- Health and wellness tips for parents of young children during COVID-19
- Resources and tips for digital learning while Philly schools are closed
- Staying healthy and seeking non-COVID-19 medical care

Where can I find more information about COVID-19?

- Philadelphia Department of Public Health
- Centers for Disease Control and Prevention

© 2022 Feuerstein-Simon R et al. *JAMA Network Open.*

Penn Medicine

DATE

To whom it may concern,

I am writing to notify you that your employee, CASE, tested positive for Covid-19 on DATE.

Our team at Penn Medicine has been in contact with CASE regarding public health guidance.

Per CDC and City of Philadelphia guidelines, employees may be able to leave isolation and return to work after meeting criteria:

- No fevers for past 24 hours without fever-reducing medication.
- Improvement of other symptoms including cough and shortness of breath.
- 10 days have elapsed since first onset of symptoms. We strongly recommend that you follow these recommendations.

For further guidance, we recommend that you consult up-to-date Philadelphia Department of Public Health guidelines, which are available at https://www.phila.gov/programs/coronavirus-disease-2019-covid-19/support-for-the-community/informationfor-businesses/.

Please do not hesitate to contact our team at covidtracing@pennmedicine.upenn.edu for further information. Thank you for all that you do to protect your employees and stop the spread of this infection.

© 2022 Feuerstein-Simon R et al. *JAMA Network Open.*

Penn Medicine

DATE

To Whom It May Concern,

Our team at Penn Medicine has determined that CONTACT has been in close contact with an individual who has tested positive for COVID-19.

Penn Medicine recommends that people who have had prolonged close contact with a person confirmed to have COVID-19 stay at home and self-isolate for 14 days after the last contact. For those people with household contacts, we recommend that people stay at home for 14 days after isolation has been discontinued for the ill member of their household.

Proof of COVID-19 testing either to qualify for sick leave or to return to work is not recommended. Per the most recent Philadelphia and CDC guidelines, employees with symptoms may return to work 1) after 10 days have passed since their symptoms first appeared and 2) at least 1 day after recovery, including resolution of fever without medications or improvement in respiratory symptoms.

For further guidance, we recommend that you consult up-to-date Philadelphia Department of Public Health guidelines, which are available at https://www.phila.gov/programs/coronavirus-disease-2019-covid-19/supportfor-the-community/information-for-businesses/.

Please do not hesitate to contact our team at CovidTracing@pennmedicine.upenn.edu for further information. We also recommend the following services or guidance:

- Penn Medicine Coronavirus Hotline at 833-983-1350
- Penn Medicine: https://www.pennmedicine.org/coronavirus/frequently-asked-questions-about-covid-19
- CDC: https://www.cdc.gov/coronavirus/2019-ncov/index.html

Thank you for all that you do to protect your employees and stop the spread of this infection.

© 2022 Feuerstein-Simon R et al. *JAMA Network Open.*

eMethods. Description of Team Roles and Responsibilities

Team Management: Team management included full-time faculty/staff for the entirety of the program, and PhD students whose research was disrupted by the pandemic from March 2020 - August 2020. Team management was instrumental in building and implementing the program, including supporting recruitment efforts, creating and deploying the training, coordinating with the university and local departments of public health, reporting to local and state officials, and overseeing all volunteer leaders, among many other tasks.

Case Investigation Team: Case investigators were responsible for investigating SARS-CoV-2+ cases, identifying their close contacts, and delivering up-to-date isolation guidance. The Case Investigation Team included 4-8 “Team Captains,” each responsible for managing groups of 5-15 volunteers (depending on case volumes and volunteer capacity), and were the first line of contact for case investigator questions. Team Captains also coordinated volunteer schedules with the Operations Team to assign cases most efficiently. For the first several months of the operation, we sought volunteers with clinical experience to serve as case investigators--although all case investigators/contact tracers were instructed not to offer clinical guidance, rather to only offer coaching on public health guidance.

Contact Tracing Team: Contact tracers were responsible for notifying close contacts of cases regarding their possible exposure and delivering up-to-date quarantine and testing guidance. The Contact Tracing Team had an identical structure to the Case Investigation Team, with a group of 4-8 “Team Captains,” as detailed above.

Operations Team: Daily, the Operations Team uploaded to REDCap case information (i.e., positive test results from the health system), as well as contact details derived from case investigations. The Operations Team worked with Team Captains regarding scheduling volunteers.

Reporting Team for High Exposure Risk Cases: This team addressed positive cases who were likely unable to maintain physical distancing, or High Exposure Risk Cases (HERC). HERCs were defined as anyone a) living or working in congregate care settings (e.g., nursing homes, shelters) or b) working in occupations with high likelihood of transmission to other people (e.g., meat processing plants). Case investigators notified the Reporting Team when they identified a HERC during an interview. Once notified of a HERC, the Reporting Team notified the appropriate local health department based on the case’s home residence.

Communications Team: Given the complexity of infection control guidance, we offered cases/contacts digital information regarding how to keep themselves and their households safe (eFigure 3). In addition to offering general follow-up

information, the Communications Team provided employer letters for any case/contact who requested one.

Social Needs Response Team: To support safe isolation/quarantine, health system faculty created a student-staffed initiative to address unmet social needs. The Social Needs Response Team (SNRT) connected patients to a range of services, including food delivery, rent assistance, medical transportation, and assistance identifying a primary care provider. Beginning in June 2020, all cases and contacts were screened for unmet social needs, and anyone that reported at least 1 unmet social need and gave consent was referred to the SNRT.

© 2022 Feuerstein-Simon R et al. *JAMA Network Open.*
